# Effect of Melt-Spinning Parameters on the Structure and Properties of Ni_55.5_Mn_18.8_Ga_24_Si_1.7_ Heusler Alloy Ribbons

**DOI:** 10.3390/ma16196590

**Published:** 2023-10-07

**Authors:** Pranav Bhale, Pnina Ari-Gur, Ronald D. Noebe, Yang Ren, Amila Madiligama, Ranjith Devaraj, Matthew S. Cook

**Affiliations:** 1 Department of Mechanical and Aerospace Engineering, Western Michigan University, Kalamazoo, MI 49008, USA; pranav.bhale@wmich.edu (P.B.);; 2 NASA Glenn Research Center, Cleveland, OH 44135, USA; ronald.d.noebe@nasa.gov; 3 Advanced Photon Source, Argonne National Laboratory, Lemont, IL 60439, USA; yangren@cityu.edu.hk; 4 Department of Physics, City University of Hong Kong, Kowloon, Hong Kong, China; 5 Department of Physics, University College Science, Pennsylvania State University, DuBois, PA 15801, USA; aum1973@psu.edu; 6 Los Alamos National Laboratory, Los Alamos, NM 87545, USA

**Keywords:** entropy, Heusler alloy, magneto-structural phase transition, magnetocaloric effect, synchrotron diffraction

## Abstract

Ni–Mn-based Heusler alloys are known to demonstrate magnetic shape memory and giant magnetocaloric effect (MCE). These effects depend on the phases, crystallographic and magnetic phase transitions, and the crystallographic texture characteristics. These structural characteristics, in turn, are a function of the processing parameters. In the current work, Ni_55.5_Mn_18.8_Ga_24_Si_1.7_ Heusler alloy was processed by melt-spinning under a helium atmosphere. This process results in a fine microstructure. The ribbon that was produced with a narrower nozzle width, faster wheel speed, and higher cast temperature, indicating a faster cooling rate, had double the magnetic entropy change close to room temperature. However, the other ribbon demonstrated a large entropy change over a broader temperature range, extending its usability. The effect of the melt-spinning process parameters on the developing microstructure, crystallographic structure and texture, transformation temperatures, and the magnetic entropy change were studied to explain the difference in magnetocaloric behavior.

## 1. Introduction

Magnetic cooling systems operating near room temperature could utilize magnetocaloric materials as a solid-state refrigerant. These materials provide an environmentally friendly alternative to conventional refrigerants used in vapor-compression refrigeration cycles [1]. The magnetocaloric effect is especially large for materials that demonstrate strong coupling between the magnetic order and crystalline phase transitions, therefore, termed the giant magnetocaloric effect (GMCE) [2]. Ni–Mn-based Heusler alloys are known to demonstrate GMCE around the phase transformation temperature [3] and hence are widely studied as candidates for magnetic refrigeration (e.g., [1,4,5]).

The higher temperature crystallographic phase in full-Heusler alloys, referred to as austenite, has a highly symmetrical crystal structure (cubic) with L2_1_, B2, or A2 ordering; L2_1_ is the most commonly found austenite structure [5]. Cooling the austenitic phase results in a solid-state phase transformation to a martensitic phase which demonstrates lower symmetry. The crystalline structure of the martensite may be orthorhombic, monoclinic, or tetragonal and often exhibits modulation [6]. In addition to a structural phase transition, Ni–Mn–Ga-based Heusler alloys belong to a group of materials known as ferromagnetic shape memory alloys (FMSMA) [7], which demonstrate a first-order magnetostructural phase transition. The austenitic phase in Heusler FMSMA is ferromagnetic below the Curie temperature, whereas the martensitic phase can have ferromagnetic, antiferromagnetic, or spin-glass magnetic order [8]. Therefore, in addition to temperature, the application of a magnetic field can affect the martensitic transformation [9,10].

Melt-spinning is a technique used to obtain ribbons with very fine grain size. The fast-cooling rate when the molten metal comes into contact with the chilled wheel greatly reduces the grain size [11]. This technique has also been utilized to obtain fine-structured and textured ribbons for applications like sensors and actuators [12,13]. Another advantage of melt spinning is that, due to the fast solidification rate, the chemical composition is homogeneous and is virtually identical to the master alloy [14,15]. Microstructural inhomogeneity and anisotropy have been known to be present in melt-spun ribbons (e.g., [13,16]). Melt-spinning parameters, such as wheel speed, melt ejection pressure/speed, nozzle dimensions, and nozzle-to-wheel distance, affect the structure, grain dimension and size, transition temperatures, and other properties of the cast ribbon [17]. Both microstructure and texture greatly affect the properties of Ni–Mn–Ga-based Heusler alloys [18]. The typical microstructure of melt-spun ribbons is columnar, perpendicular to the ribbon surface [15,19,20,21]. This microstructure leads to the formation of fiber texture. In the melt-spun ribbon, often the fiber texture reported is [220] [14,20], although Li [22] found [004] texture. Annealing, results in recrystallization which causes the rotation to [004] fiber texture perpendicular to the surface [14].

The work presented here focuses on the effects of several manufacturing parameters of the melt-spinning process on the structure, phase transformations, and magnetocaloric properties of Ni–Mn–Ga-based melt-spun ribbons.

## 2. Materials and Methods

A polycrystalline alloy master ingot was cast by arc-melting a Ni–Mn master alloy with Ga and Si additions. Starting with the Ni–Mn master alloy, instead of using elemental Mn, and given the short time the material was molten, Mn evaporation and loss was minimal. All melting was performed starting with elemental constituents purchased from Stanford Advanced Materials (Lake Forest, CA, USA). The aim was a composition of Ni_56.2_Mn_18.8_Ga_23.2_Si_1.8_ (all compositions given are atomic percent). The pre-alloyed ingot was then induction-melted and melt-spun under a helium atmosphere using a Buehler HV melt-spinner (Bodelshausen, Germany) using BN nozzles. Two different sets of parameters were used to produce two variants of ribbons (IDs: BMS220 and BMS222, see Table 1). The BMS220 and BMS222 ribbons were approximately 30 μm and 20 μm thick, respectively, due to the faster wheel speed and thus, faster cooling rate for the latter. The final composition and melt-spinning parameters are summarized in Table 1. A SPECTRO Analytical Instruments (Kleve, Germany) ARCOS MV inductively coupled plasma-atomic emission spectroscopy (ICP-AES) system was used to determine the final chemical compositions of the ribbons listed in Table 1, which is essentially indistinguishable from the aim composition even with the small uncertainty in the ICP-AES measurements (approximately ±1% of the absolute value being measured). The melt-spun ribbons have two distinct surfaces: one in contact with the water-cooled copper wheel (‘wheel-side’) and the other in contact with the helium atmosphere (‘free-side’). The two sides experience significantly different cooling rates, with the wheel-side cooling faster.

The microstructure of the ribbon’s cross-sections was studied using a JSM-7200F (Akishima-shi, Japan) Schottky field emission scanning electron microscope (FESEM). High-resolution images were obtained using the retractable backscattered electron detector (RBED) with a low acceleration voltage of 5 kV. To obtain a high-quality polished surface, the final polishing step was carried out using a Buehler VibroMet^TM^ 2 (Lake Bluff, IL, USA) vibratory polisher using a colloidal silica polishing suspension, followed by etching in 5% Nital solution. Energy-dispersive X-ray spectroscopy (EDX) on the cross-sections was used to confirm the chemical composition uniformity.

**Figure 1:**
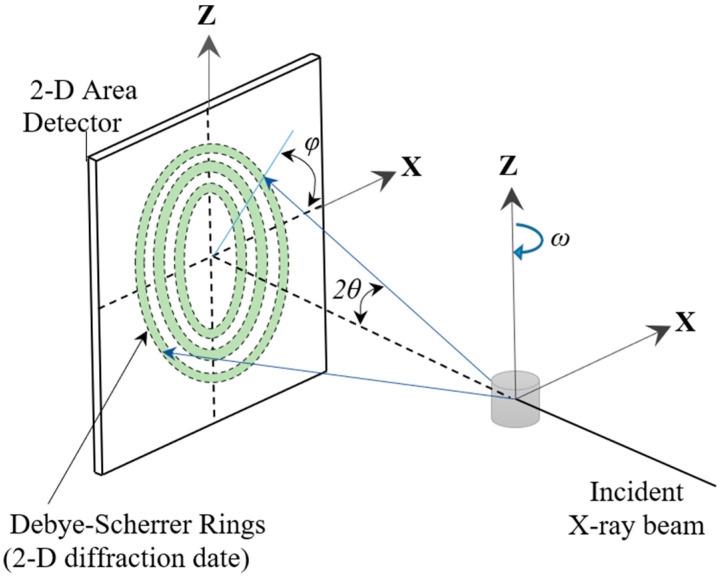
Schematic diagram for synchrotron X-ray diffraction (adapted from [23]). The X-ray beam incident to the sample diffracts at an angle 2θ. The diffracted Debye rings are captured by the 2D area detector, and the results are saved as 2D images.

For preliminary phase analysis, ambient-condition X-ray diffraction was performed using CoKα radiation (λ = 0.1790 nm) on a Malvern Panalytical Empyrean X-ray diffractometer. For crystal structure and crystallographic texture analyses, synchrotron diffraction was carried out at the 11-ID-C beamline of the advanced photon source (APS), Argonne National Laboratory, using a high-energy beam (λ = 0.01173 nm). Cerium dioxide (CeO_2_) powder was used as a reference to determine the instrument parameters. Figure 1 shows a schematic diagram of the 2D image of the diffracted patterns, with the Debye rings, sample rotation angle (*ω*), and azimuthal angle (*η*) defined. This experimental setup to obtain the 2D image of the diffracted patterns of the Debye rings was similar to the one described by Lutterotti et. al. [23]. The 2D images were integrated using the general structure and analysis software (GSAS-II) version 4234 [24] to obtain 1D patterns, refine the structure, and construct pole figures using the spherical harmonics model developed by Von Dreele [25]. For all the ribbons, the cylindrical (fiber texture) model was assumed in GSAS-II as a starting point in the refinement process because fiber texture normal to the plane of the ribbon is typically observed in melt-spun ribbons of polycrystalline alloys, e.g., in Fe–Si [26], Cu–Al–Ni- [27], and Ti–Ni–Cu-based alloys [13].

Quantum design-physical property measurement system-vibrating sample magnetometer (QD-PPMS-VSM) was used to measure the isotherms of the magnetic moment (*m*) as a function of the magnetic field (*H*). The field was changed from 0 to 14 T at temperatures of 260 to 335 K (in 3 K steps). The magnetization (*M*) values were then calculated by dividing the magnetic moment by the sample mass. The magnetization as a function of field and temperature was used to estimate the change in entropy using Maxwell’s equations [28,29]. The magnetization results were also used to determine the Curie temperature.

Resistivity measurements as a function of temperature and magnetic field were determined using the QD-PPMS on samples measuring 4 × 1.5 × 0.03 and 2.8 × 2 × 0.03 mm^3^, respectively. The magnetic field range varied from 0 to 11 T. The calculated resistivity was normalized with respect to the maximum resistivity for each variant (at 2 T for BMS220 and at 0 T for BMS222). The results were used to determine the transformation temperatures of the samples as a function of the magnetic field.

## 3. Results

### 3.1. Microstructure

**Figure 2:**
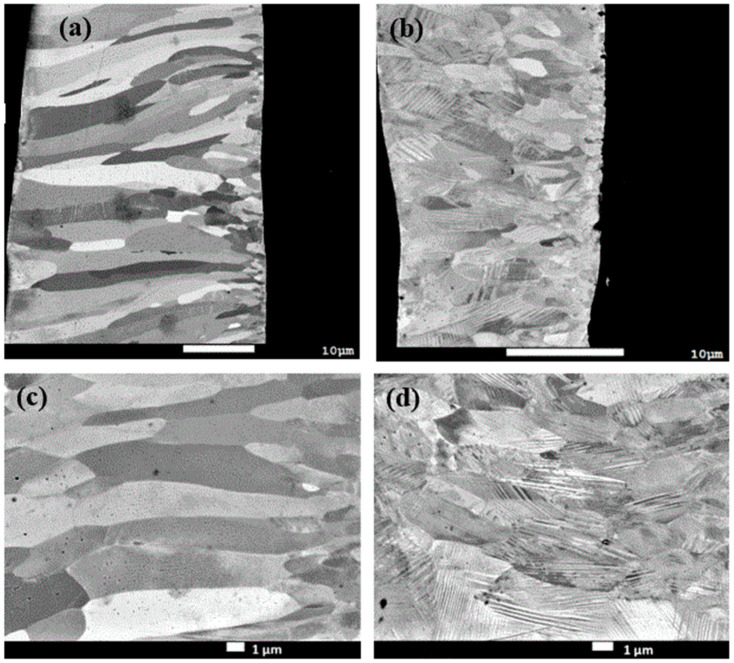
SEM images of cross-sections of BMS220 and BMS222 as-melt-spun ribbon. (**a**,**b**) Cross-sections of BMS220 and BMS222, respectively. (**c**,**d**) Higher resolution images of (**a**,**b**), respectively. The right side of each image is the wheel-side of the ribbon, and the left side is the free-side.

Scanning electron microscopy observations on the cross-sections of BMS220 and BMS222 are shown in Figure 2. Small, equiaxed grains can be seen along the right side of Figure 2a,b (wheel-side). Then, long columnar grains grow from the wheel-side toward the free-side so that most of the cross-section of the ribbons consists of anisotropic columnar grains. Martensitic twins are clearly visible on the BMS222 cross-section. BMS220 is about 30 μm thick, while BMS222 has an uneven thickness of about 20 μm resulting from a faster wheel speed and narrower nozzle width.

### 3.2. Crystallography and Phase Analysis

**Figure 3:**
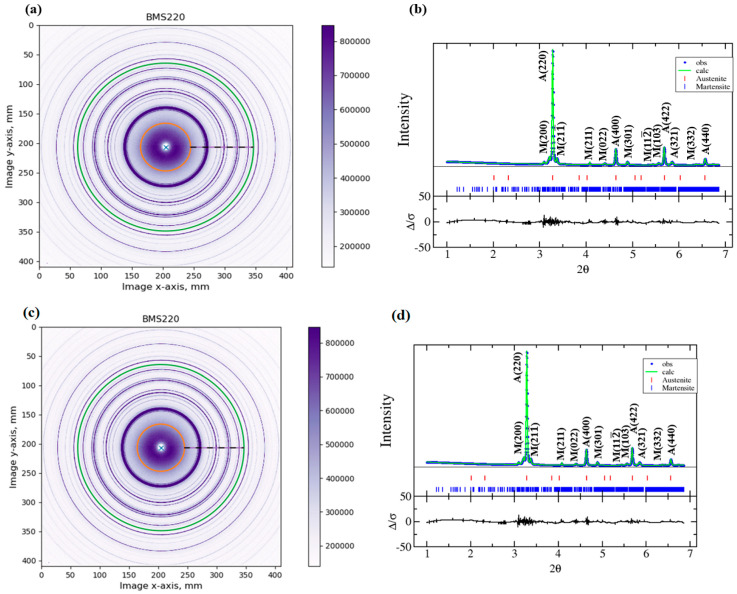
Synchrotron results were presented as calibrated 2D images for BMS220 (**a**) and BMS222 (**c**). The refined, integrated pattern for austenite and martensite is shown for BMS220 (**b**) and BMS220 (**d**).

Ambient synchrotron diffraction patterns of BMS220 and BMS222 were calibrated and refined (Figure 3) to determine the crystal structures of the austenitic and martensitic phases. The values reported in [30] were used as initial inputs in GSAS-II for the crystal structure and parameters of the martensite phase. They were then refined to obtain the crystal structure of the martensite in both ribbons. The refined values of the lattice parameters of BMS220 and BMS222 were similar. The austenitic phase was cubic with *L2_1_* order (*Fm*$\bar{3}$*m* space group) with a lattice parameter of 0.5786 (5) nm. Whereas the martensitic phase was monoclinic and belonged to the *I2/m* space group with refined lattice parameters of a = 0.4190 (7) nm, b = 0.5492 (6) nm, c = 0.4248 (6) nm and *β* = 93.388°. The modulation of the martensite was studied using the superspace theory [31], in which an extra dimension is added to define the aperiodicity in the atomic positions as a periodic occurrence. The analysis of this periodicity requires an assumption of a modulation wave vector (*q*) defined by Equation (1) [32], where the components ($p_{1},\;p_{2},\;p_{3}$) of the basis vectors in the reciprocal lattice ($a^{*}$, $b^{*}$, $c^{*}$) determine the magnitude of the modulation wave vector.

(1) $$q=p_{1}a^{*}+p_{2}b^{*}+p_{3}c^{*}$$

In the determination of *q*, it was assumed that the modulation occurs along the [001] martensitic direction. This is because of the Bain distortion in the martensitic transformation, where the <110> direction in austenite is parallel to the [001] direction of the martensite phase [33]. The initial values of $p_{1}$ and $p_{2}$ were assumed to be zero, whereas $p_{3}$ was assumed to be 2/7, based on its value in the incommensurate structure of 7M modulated martensite in similar alloys [34]. The refined modulation vector was found to be *q* = 0.305c*. The superspace group for the martensite was then determined to be *I2/m*(*α0γ*)*00*. A similar superspace group was observed in the Ni_53.75_Mn_21.25_Ga_25_ bulk alloy [30]. The value of $p_{1}$ was almost zero, and the value of $p_{3}$ is sufficiently close to 2/7. Hence, the crystal structure of martensite was determined as incommensurate 7M modulated.

The room-temperature phase fractions in BMS220 were determined to be 58% austenite and 42% martensite. The synchrotron experiment was conducted in transmission mode, so the phase fractions obtained from the refinement of the diffraction results represent an average across the sample thickness. Grain sizes of both phases were obtained from Rietveld refinement of the synchrotron diffraction patterns. The grain size for the austenite and martensite were determined to be 0.950 ± 0.007 and 0.900 ± 0.004 μm, respectively. It should be noted, though, that the process is highly anisotropic, and as a result, the grains are not generally equiaxed, as seen in Figure 2. Thus, the results best represent a combination of the equiaxed grain size along the wheel-side of the ribbon and the columnar grain diameter.

The phase fractions in BMS222 were determined to be 22% austenite and 78% martensite. This ratio is significantly different from that found in BMS220 at room temperature, which was 58% austenite and 42% martensite. This conclusion confirms the observation from the SEM results (Figure 2) that clearly show the martensitic twins for most of the cross-section of the BMS222 sample. The austenite and martensite grain sizes (0.420 ± 0.003 and 0.340 ± 0.005 μm, respectively) in BMS222 were almost half the size of the grains in BMS220 due to the higher cooling rate. However, as previously mentioned, the grain size determined by synchrotron diffraction does not consider the significant microstructural anisotropy that is evidenced in Figure 2.

### 3.3. Ambient X-ray Diffraction

**Figure 4:**
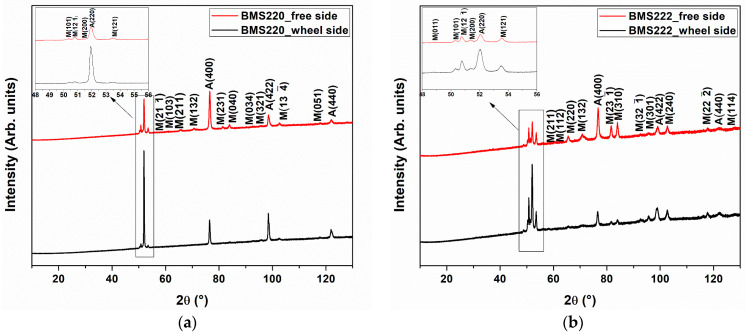
Ambient XRD patterns for free- and wheel-side of BMS220 (**a**) and BMS 222 (**b**) melt-spun ribbon. Peaks are indexed for austenite (A) and martensite (M) phases.

The two sides of the ribbons (free- and wheel-side) experience different cooling rates. To evaluate the effect of this difference on structure, room temperature X-ray diffraction of each side of the ribbons was performed, and the patterns are shown in Figure 4 for BMS220 (a) and BMS222 (b), respectively. At room temperature, austenite and martensite co-exist on both the free- and wheel-side of BMS220 and BMS222. However, the fraction of austenite on the wheel-side was considerably higher compared to the free-side, as observed from the intense wheel-side austenite diffraction peaks in Figure 4. This may be a result of the faster cooling rate close to the wheel-side surface.

### 3.4. Crystallographic Texture

The study of the crystallographic texture present in the ribbons was performed to assess the impact of the melt-spinning process parameters on developing anisotropy. The texture analysis was based on the ambient synchrotron diffraction patterns. Because of the nature of melt-spinning, where solidification starts from the melt contact with the chilled wheel and progresses through the thickness toward the free surface, as observed also in the columnar microstructure (Figure 2), the starting texture model in GSAS-II, was assumed to be cylindrical (fiber texture). This assumption was also based on other known cases of the nature of the texture in melt-spun ribbons, e.g., [13,19].

#### 3.4.1. Texture of the BMS220 Ribbon

**Figure 5:**
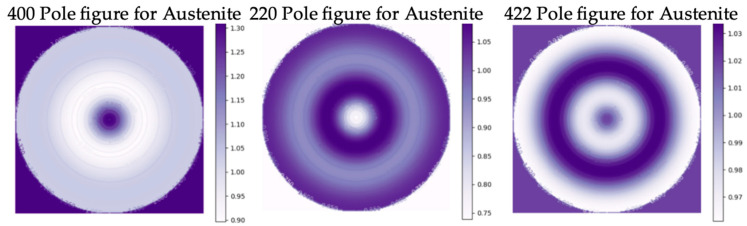
400, 220, and 422 pole figures of austenite in BMS220 melt-spun ribbon.

**Figure 6:**
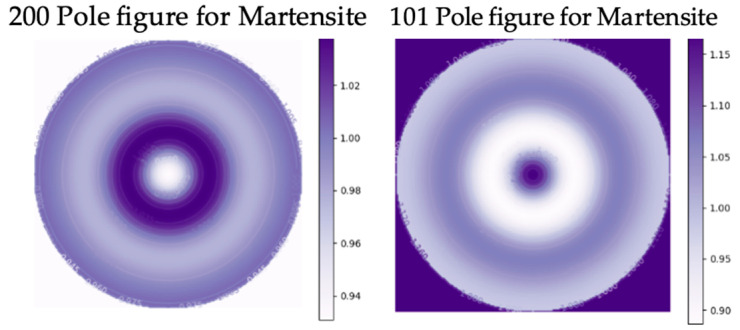
200 and 101 pole figures of martensite in BMS220 melt-spun ribbon.

The pole figures of the austenite and martensite of the BMS220 melt-spun ribbon revealed a weak fiber texture (Figure 5 and Figure 6). In the austenite, the <200>_A_ fiber direction was oriented parallel to the ribbon’s normal direction along the columnar grain. Pole figures of the martensite reveal [101] fiber texture parallel to the ribbon’s normal direction. This results from the directional relationship between the phases in martensitic transformation [33]. The notations used for the Miller indices were <uvw> for cubic austenite (family of directions) and [uvw] for monoclinic martensite (directions).

#### 3.4.2. Texture of the BMS222 Ribbon

**Figure 7:**
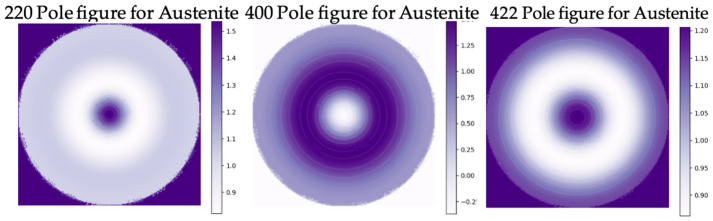
220, 400, and 422 pole figures of austenite planes in BMS222 melt-spun ribbons.

**Figure 8:**
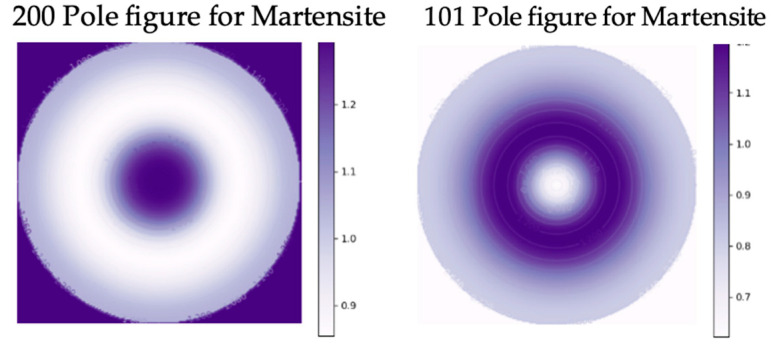
200 and 101 pole figures of martensite planes in BMS222 melt-spun ribbons.

The fiber texture of the BMS222 ribbons for both the austenite and martensite (Figure 7 and Figure 8) was different from that of BMS220 in both intensity and nature. The BMS222 texture was stronger, and, in the austenite, the <110>_A_ fiber was oriented parallel to the normal to the ribbon (90 degrees to the <200>_A_ fiber in BMS220). Directional relationship during the martensitic transformation [33] resulted in a [200]_M_ fiber texture parallel to the normal direction in the ribbon.

Texture has an impact on the magnetic behavior of Heusler alloys. For example, it was reported that strong preferred orientation along [110] in body-centered tetragonal (bct) martensite in Ni_54_Mn_21_Ga_25_ melt-spun ribbons was responsible for high magnetization values (around 60 emu/g) [35]. The [110] direction in bct martensite is parallel to the <100> direction of the cubic austenite phase. In the current study, the texture of BMS222 could have contributed to its higher magnetization values.

#### 3.4.3. Magnetic Behavior of the Ribbons

**Figure 9:**
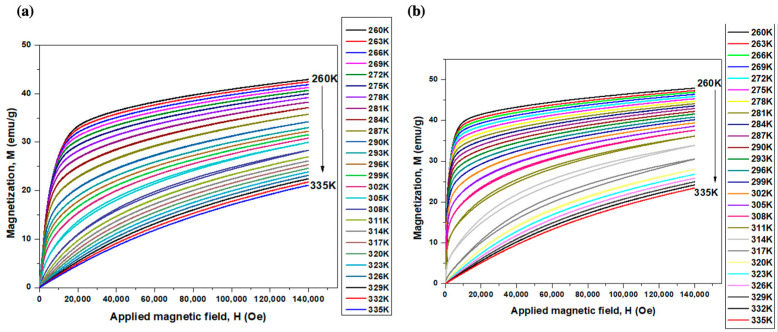
Isothermal magnetization curves for (**a**) BMS220 and (**b**) BMS222 melt-spun ribbon.

Isothermal magnetization curves of BMS220 (Figure 9a) and BMS222 (Figure 9b) melt-spun ribbons show the dependency of magnetization (M) on the applied magnetic field (H) at different temperatures. It can be observed that BMS222 had higher values of magnetization compared to BMS220; the maximum magnetization for both BMS220 (42 emu/g) and BMS222 (49 emu/g) was observed at 260 K, the lowest temperature investigated, under an applied field of 140 kOe.

**Figure 10:**
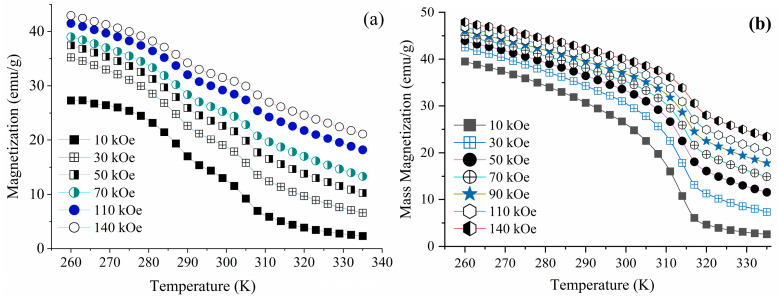
Mass magnetization vs. temperature of BMS220 (**a**) and BMS222 (**b**) at selected applied fields.

The magnetization results were also used to determine the Curie temperature of each ribbon. The graphs showing the mass magnetization vs. temperature of BMS220 and BMS222 at selected applied fields are presented in Figure 10a,b respectively. From the graphs, the Curie temperature was estimated to be T_c_ = 305 K for BMS220 and T_c_ = 315 K for BMS222.

#### 3.4.4. Martensitic Transformation Temperatures

**Figure 11:**
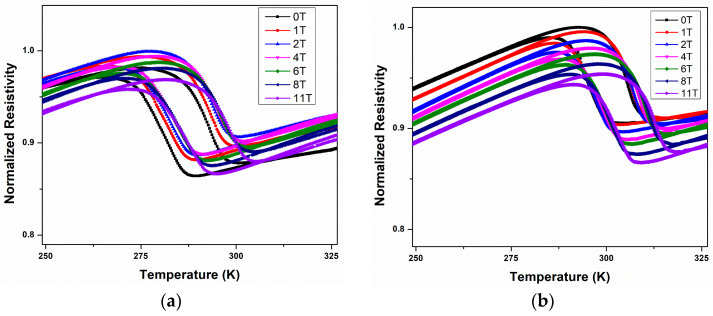

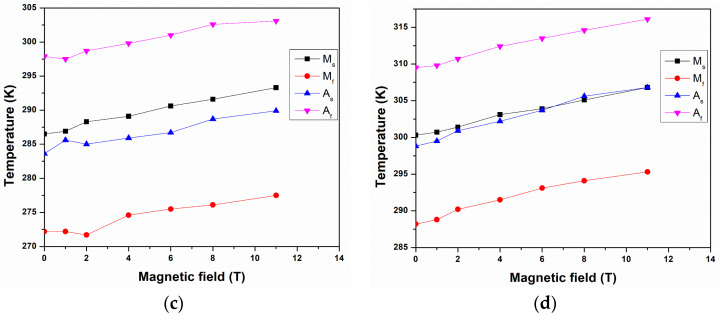
Temperature dependence of the resistivity under applied magnetic fields in the range of 0 to 11 T for BMS220 (**a**) and BMS222 (**b**). Martensitic transformation temperature as a function of the magnetic field, obtained from the resistivity curves for BMS220 and BMS222, as depicted in (**c**) and (**d**), respectively.

To determine the martensitic transformation temperatures, the resistivity as a function of temperature and magnetic field (Figure 11a for BMS220 and Figure 11b BMS222) was used. The martensitic transformation temperatures were determined using the point of inflection of cooling and heating cycles, and the temperatures replotted as a function of the magnetic field in Figure 11c,d to demonstrate the effect of the application of magnetic field on the transformation temperatures for BMS220 and BMS222, respectively. At all fields, the transformation temperatures of BMS222 were higher than those of BMS220. Also, the trend demonstrates an increase in the transformation temperature with the increasing applied field, with the effect of the field stronger for the BMS222 ribbons, opening the way for more control.

## 4. Discussion

The crystallographic phases, magnetic and structural phase transformations, and crystallographic texture are all critical properties in determining the performance of materials for magnetocaloric applications.

### 4.1. Crystallographic Phases

Room temperature crystallographic phases and textures of the melt-spun ribbons are summarized in Table 2.

It can be seen that the different processing routes lead to the formation of the same crystallographic structures for both the austenite and martensite. This is important because the change in entropy during the martensitic phase transformation contributes to the GMCE. The noticeable difference in the phase fractions at room temperature between BMS220 and BMS222 (also evident in the SEM images, Figure 2) stems from the difference in the transformation temperatures (see the following discussion), which is process dependent.

Crystallographic texture may play a role in magnetic refrigeration. In the current study, even though both textures were not very strong, the <110>_A_ fiber texture of BMS222 is sufficiently meaningful to result in higher magnetization values compared to BMS220 with a weak <200>_A_ texture. The presence of different fiber texture variants is interesting, and its dependence on the process parameters justifies further consideration in the development of the melt-spinning process.

### 4.2. Phase Transformation Temperatures

Phase transformations are the driving force behind the giant magnetocaloric effect. The temperatures at which they occur and whether the magnetic and crystallographic transformations merge play an important role in the determination of the GMCE, its magnitude, and the range of efficacy. The crystallographic and magnetic transformation temperatures found in the melt-spun ribbons are summarized in Table 3.

Both ribbons demonstrate a martensite-start *M_s_* (K) point at a higher temperature than austenite-start *A_s_* (K), a relatively less common phenomenon. This results from the broad transformation range. The magnetic transformation temperature *T_c_* (K) lies very close to *A_f_* (K). This is beneficial for the magnetocaloric effect, as the entropy change in the two transformations adds up to produce a larger effect.

### 4.3. Magnetocaloric Effect

The isothermal magnetization curves shown in Figure 9 for BMS220 (a) and BMS222 (b) were used to estimate the magnetocaloric effect by calculating the change in magnetic entropy, $∆S_{m}\left(T,\;H\right)$.

Maxwell’s equation (Equation (2)) was used to calculate the change in magnetic entropy from the isothermal magnetization results [36]. Equation (2) was numerically integrated using the trapezoidal method in the Origin^®^ software version 8.5 to obtain $∆S_{m}\left(T_{av},\;H\right)$ (Equation (3)), where $T_{av}$ is defined as $T_{av}=(T_{i+1}+T_{i})/2$.

(2) $$∆S_{m}\left(T,\;H\right)=\int_{0}^{H}{\left(\frac{\partial M}{\partial T}\right)}_{H}dH$$

(3) $$∆S_{m}\left(T_{av},\;H\right)=\sum_{i=1}^{i_{max}}\frac{M\left(T_{i+1},H_{i}\right)-M(T_{i},H_{i})}{T_{i+1}-T_{i}}\left(H_{i+1}-H_{i}\right)$$

**Figure 12:**
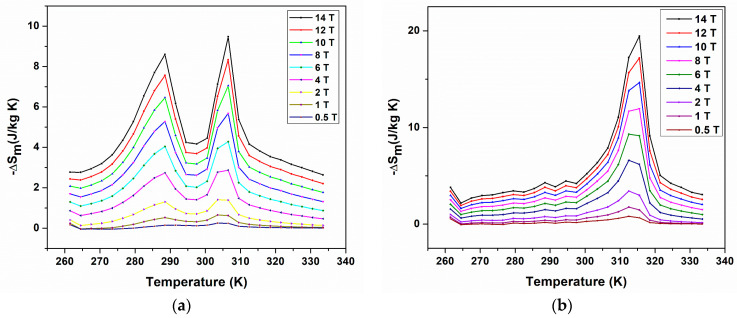
Temperature dependence of the magnetic entropy change for BMS220 (**a**) and BMS222 (**b**) melt-spun ribbons under magnetic fields H = 0.5 to 14 T.

It can be observed from the M (H) curves of BMS220 and BMS222 (Figure 9a,b) that the magnetization decreased with an increase in temperature. BMS222 had higher values of magnetization compared to BMS220, where the maximum magnetization for both BMS220 (42 emu/g) and BMS222 (49 emu/g) was observed at 260 K and 140 kOe. The $∆S_{m}\left(T\right)$ curve for BMS220 (Figure 12a) had two distinct peaks of ~8.5 and 9.5 J/kg K at 288 and 306 K, respectively. In contrast, a single peak at 318 K was observed in $∆S_{m}\left(T\right)$ curve for BMS222 (Figure 12b). The presence of two distinct peaks at different temperatures for BMS220 is additional confirmation that the structural phase transition (at 288 K) and magnetic phase transition (at 306 K) occur at different temperatures. The structural phase transition temperature for BMS220, determined by the change in resistivity, was 288 K. These values are consistent with the first peak observed in the temperature-dependent magnetic entropy change observed in Figure 12a. The $∆S_{m}\left(T\right)$ curve for BMS220 also indicates that both martensite and austenite are ferromagnetic, and austenite becomes paramagnetic at 306 K. In BMS222, a coupled magneto-structural phase transition around 318 K resulted in a $∆S_{m}$ value of more than twice that of BMS220. However, the broader temperature range for the entropy change in BMS220 presents an opportunity to expand the temperature range where the magnetocaloric effect is present. The lower symmetry in the crystal structure of martensite in Ni–Mn–Ga alloys improves the magneto-structural anisotropy [37], which makes magnetization saturation easier in the more symmetric crystal structure of austenite [38].

## 5. Conclusions

Melt-spinning of a Ni_55.5_Mn_18.8_Ga_24_Si_1.7_ Heusler alloy, using two different processing parameter sets, resulted in ribbons that differed significantly in both structure and properties. The BMS222 ribbon, when compared to BMS220, was produced with a narrower nozzle width, faster wheel speed, and higher cast temperature, resulting in a faster solidification rate. As a result, BMS222 had double the magnetic entropy change close to room temperature compared to BMS220. This difference in magnetic behavior was, in turn, traced to the difference in magnetic and structural transformations in the alloys, with the exceptional results for BMS222 due to combined magnetostructural transitions occurring at essentially the same temperature. Conversely, it should be noted that although a large magneto-entropy change is desired, the smaller effect in the BMS220 ribbon was spread over a broader temperature range, increasing its range of usability.

The crystallographic texture of the ribbons was studied because texture may play a role in magnetic refrigeration. Fiber texture was found in both cases. In the BMS220, a weak texture with the <200>_A_ fiber direction of the austenite, oriented parallel to the ribbon’s columnar grains; it transforms to [101]M in the martensite due to the directional relationship in the martensitic transformation [33]. The BMS222 texture was somewhat stronger, with <110>_A_ fiber texture in the austenite and [200]M in the martensite; both are 45 degrees to the fiber texture in BMS220. The effect of process parameters on the different fiber texture variants is interesting and incites further development of the melt-spinning process. In the current study, the higher magnetization in the BMS222 ribbon could be attributed to its crystallographic texture.

Process parameters influenced the crystallographic transformation temperatures, with a faster cooling rate due to narrower nozzle width, faster wheel speed, and higher cast temperature for BMS222, resulting in higher transformation temperatures and a stronger trend of further increase in the transformation temperature with an applied magnetic field. This, too, opens the way to more control in optimizing materials for solid-state refrigeration, though a significant amount of work is needed to understand the complete range of processing–structure relationships in these materials.

## Figures and Tables

**Table 1:** Composition and melt-spinning parameters.

Ribbon ID	Chemical Composition (ICP-AES) (at. %)	Cast Temperature (°C)	Nozzle Width (mm)	Wheel Speed (m/s)	Avg. Ribbon Thickness (μm)
BMS220	Ni_55.4_Mn_18.9_Ga_24_Si_1.7_	1300	17	15.7	30
BMS222		1350	3	19.6	20

**Table 2:** Room temperature crystallographic phases and texture.

Sample ID	Crystal Structure	Site Occupancy	Phase Fraction (%)	Texture
BMS220	L2_1_ austenite (cubic)	***8c***: Ni; *4b*: Ga, Si; ***4a***: Mn, Ni, Si	57	Fiber: Austenite <200>_A_ Martensite [101]_M_
	7M modulated martensite (monoclinic)	***4h***: Ni; ***2d***: Ga, Si; ***2a***: Mn, Ni, Si	43	
BMS222	L2_1_ austenite (cubic)	***8c***: Ni; ***4b***: Ga, Si; ***4a***: Mn, Ni, Si	22	Fiber: Austenite <110>_A_ Martensite [200]_M_
	7M modulated martensite (monoclinic)	***4h***: Ni; ***2d***: Ga, Si; ***2a***: Mn, Ni, Si	78	

**Table 3:** Crystallographic and magnetic transformation temperatures.

Ribbon ID	*M_s_* (K)	*M_f_* (K)	*A_s_* (K)	*A_f_* (K)	*T_c_* (K)
BMS220	286.5	272.2	283.6	298.0	305
BMS222	300.3	288.2	298.8	309.5	315

## Data Availability

Not applicable.
